# Experimental Realization of a Quantum Pentagonal Lattice

**DOI:** 10.1038/srep15327

**Published:** 2015-10-15

**Authors:** Hironori Yamaguchi, Tsuyoshi Okubo, Shunichiro Kittaka, Toshiro Sakakibara, Koji Araki, Kenji Iwase, Naoki Amaya, Toshio Ono, Yuko Hosokoshi

**Affiliations:** 1 Department of Physical Science, Osaka Prefecture University, Osaka 599-8531, Japan; 2 Institute for Solid State Physics, The University of Tokyo, Chiba 277-8581, Japan; 3 Department of Applied Physics, National Defense Academy, Kanagawa 239-8686, Japan

## Abstract

Geometric frustration, in which competing interactions give rise to degenerate ground states, potentially induces various exotic quantum phenomena in magnetic materials. Minimal models comprising triangular units, such as triangular and Kagome lattices, have been investigated for decades to realize novel quantum phases, such as quantum spin liquid. A pentagon is the second-minimal elementary unit for geometric frustration. The realization of such systems is expected to provide a distinct platform for studying frustrated magnetism. Here, we present a spin-1/2 quantum pentagonal lattice in the new organic radical crystal *α*-2,6-Cl_2_-V [=*α*-3-(2,6-dichlorophenyl)-1,5-diphenylverdazyl]. Its unique molecular arrangement allows the formation of a partially corner-shared pentagonal lattice (PCPL). We find a clear 1/3 magnetization plateau and an anomalous change in magnetization in the vicinity of the saturation field, which originate from frustrated interactions in the PCPL.

Closed loop lattice systems with an odd number of antiferromagnetic (AFM) bonds induce frustration through competing exchange interactions that cannot be simultaneously satisfied. Pentagonal lattices can therefore induce frustration in analogy with systems based on triangular lattice, which have been investigated extensively^1^,^2^,^3^,^4^,^5^,^6^,^7^. Quantum pentagonal systems have yet to be realized experimetaly however. Regular pentagons cannot tile a plane because of the crystallographic restriction theorem, such that distortion and/or additional shapes are necessary^8^,^9^,^10^,^11^. The Cairo pentagonal lattice—a two-dimensional plane consisting of distorted pentagons with two inequivalent sites^8^—has attracted considerable attention since its recent realization in iron-based compounds with classical spins^12^,^13^. Although the lattice systems in iron-based compounds differ somewhat from the regular Cairo pentagonal lattice, their realizations have inspired further theoretical studies on quantum cases^14^,^15^,^16^,^17^, where the emergence of a spin-nematic phase and a 1/3 magnetization plateau are predicted. These specific quantum phases originate from the two types of inequivalent sites and the six spins in the magnetic unit cell, important characteristics that are common to the Cairo pentagonal lattice and the partially corner-shared pentagonal lattice (PCPL) investigated here.

Here, we introduce the basic properties of the PCPL. It contains two inequivalent sites, *α* and *β*, with coordination numbers 2 and 4, respectively, as shown in Fig. 1(a). The *α* site has two *β* neighbors, whereas each *β* site is connected to one *α* and three *β* sites. One ferromagnetic (FM) interaction *J*_1_ and two AFM interactions *J*_2_ and *J*_3_ form a twisted pentagonal unit consisting of *J*_1_-*J*_3_-*J*_2_-*J*_3_-*J*_1_ and induce frustration. The unit cell of the PCPL contains two *α* and four *β* sites (see Supplementary Information). The six spins in the unit cell and the observed 1/3 magnetization plateau suggest the existence of a nonmagnetic singlet state with an excitation energy gap formed by the AFM interactions *J*_2_ and/or *J*_3_ between the *β* sites. The residual *α*-site spins interact with one another through the triplet excited states of such singlet state.

The symmetry and shape of electron orbitals make crystals based on pentagonal lattices difficult to form in inorganic materials. In fact, there are few examples in the history of condensed matter physics. Unconventional lattice system should however be realizable with organic radical materials in diverse molecular arrangements. We recently established synthetic techniques for the preparation of high-quality verdazyl radical crystals^18^. In contrast to other conventional radicals such as nitroxide and nitronyl nitroxide, the *π*-electron spin density of verdazyl radicals can be delocalized even in non-planar molecular structures. This makes the molecular orbitals (MOs) associated with exchange interactions flexible in shape and enables the design of lattice systems by chemical modification, thereby facilitating the synthesis of new materials forming unconventional lattice systems^18^,^19^,^20^,^21^. Here, we present the successful synthesis of a new verdazyl radical crystal, *α*-2,6-Cl_2_-V. *Ab initio* MO calculations indicate the formation of an *S* = 1/2 quantum pentagonal lattice consisting of one FM and two AFM interactions.

## Results and Discussion

**Figure 1:**
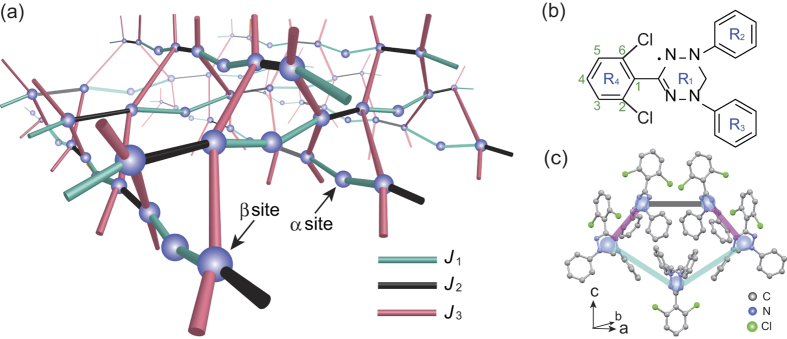
Magnetic and crystal structures of *α*-2,6-Cl_2_-V. (**a**) Partially corner-shared pentagonal lattice (PCPL) of the *α*-2,6-Cl_2_-V crystal. The blue sphere and the three types of solid lines denote spin-1/2 and intermolecular interactions *J*_1_, *J*_2_, and *J*_3_, respectively. There are two inequivalent sites, *α* and *β*, with coordination numbers 2 and 4, respectively. The two *J*_1_-*J*_1_-*J*_2_ chains are running along the *a* + *b* and *a* − *b* directions, and *J*_3_ corresponds to the interchain interaction. Thus, the unit cell includes six sites consisting of two *α* and four *β* sites. (**b**) Molecular structure of 2,6-Cl_2_-V. The central verdazyl ring with four nitrogen atoms and three phenyl rings are labeled R_1_, R_2_, R_3_, and R_4_, respectively. The Cl atoms introduced at the 2,6-position induces a relatively large dihedral angle (>80°) only in R_1_-R_3_ owing to the electrostatic repulsion between the Cl and N atoms. (**c**) Crystal structure of the twisted-plane pentagonal unit. The hydrogen atoms are omitted for clarity. The non-planar structure of the molecules inhibits simple stacking, resulting in the formation of an unconventional pentagonal unit.

Figure 1(b) shows the molecular structure of 2,6-Cl_2_-V. The crystallographic parameters at room temperature are as follows^22^: orthorhombic, space group *Fdd*2, *a* = 42.759(3) Å, *b* = 15.5551(12) Å, *c* = 16.6127(13) Å, *V* = 11049.4(15) Å^3^, *Z* = 24, *R* = 0.0346, and *R*_*w*_ = 0.0846. Furthermore, there is no indication of a structural phase transition down to around 23 K (see Supplementary Table S1). The central verdazyl ring with four nitrogen atoms and three phenyl rings are labeled R_1_, R_2_, R_3_, and R_4_, respectively [Fig. 1(b)]. The crystals contain two crystallographically independent molecules, in which the large ionic radius of the Cl atom, introduced at the 2,6-position, induces a relatively large dihedral angle (>80°) at R_1_-R_3_ owing to electrostatic repulsion between the Cl and N atoms [Fig. 1(b)]. This non-planar structure inhibits molecular stacking and the overlap of singly occupied MOs, resulting in the formation of the PCPL.

*Ab initio* MO calculations on the basis of the crystallographic arrangement at 23 K show that one FM and two AFM exchange interactions, labeled as *J*_1_, *J*_2_, and *J*_3_ (see Supplementary Fig. S2), are dominant. They are evaluated as *J*_1_/*k*_B_ = −1.6 K, *J*_2_/*k*_B_ = 3.9 K, and *J*_3_/*k*_B_ = 3.3 K, which are defined in the Heisenberg spin Hamiltonian given by 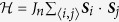, where 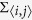 denotes the sum over the neighboring spin pairs. The three evaluated interactions form a twisted pentagonal unit consisting of *J*_1_-*J*_3_-*J*_2_-*J*_3_-*J*_1_, as shown in Fig. 1(c). The pentagonal units are connected to one another by sharing a corner, resulting in the formation of the *S* = 1/2 PCPL, as shown in Fig. 1(a).

**Figure 2:**
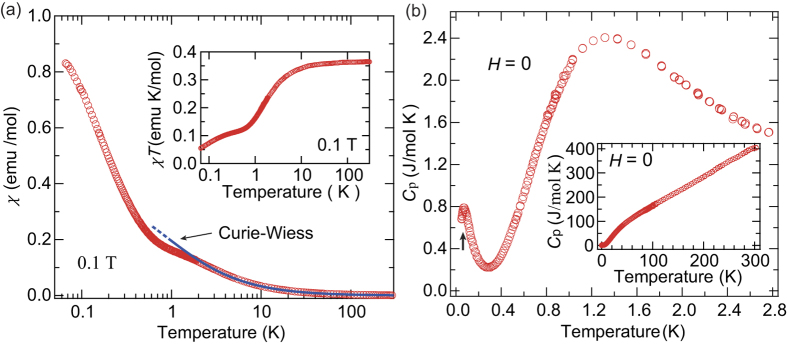
Temperature dependence of the magnetization and specific heat of *α*-2,6-Cl_2_-V. (**a**) Temperature dependence of the magnetic susceptibility (*χ* = *M*/*H*) of *α*-2,6-Cl_2_-V at 0.1 T . A shoulder appears in *χ* at about 1 K with a corresponding two-step decrease in *χ*Τ. The solid line indicates the Curie-Weiss law. (**b**) Temperature dependence of the total specific heat (*C*_p_) of *α*-2,6-Cl_2_-V at zero-field. The inset shows *C*_p_ up to 300 K. A peak characteristic of Schottky-like behavior appears at about 1.4 K. The arrow indicates a sharp peak associated with the phase transition to an ordered state at about 0.07 K.

Figure 2(a) shows the temperature dependence of the magnetic susceptibilities (*χ* = *M*/*H*) at 0.1 T. At temperature above 50 K, the Curie-Weiss law is followed, *χ* = *C*/(*T* − *θ*_W_). The estimated Curie constant is about *C* = 0.362 emu· K/mol, which is close to the expected value for noninteracting *S* = 1/2 spins, and the Weiss temperature is estimated to be *θ*_W_ = −0.85(5) K. Considering the mean-field approximation expressed as 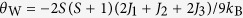, this small absolute value of *θ*_W_ indicates a weak internal field due to competition between the FM and AFM interactions. We observe a shoulder and corresponding two-step decrease in *χ*Τ at about 1 K [inset of Fig. 2(a)] , which indicate the contribution of two or more types of AFM interactions.

**Figure 3:**
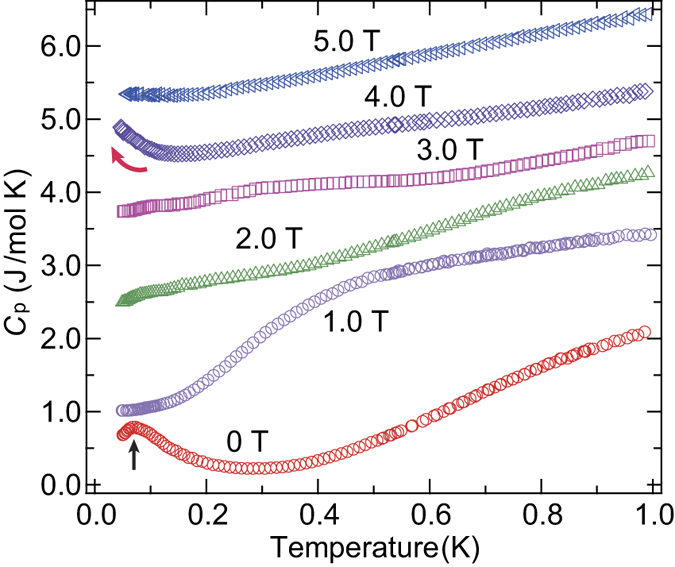
Low-temperature specific heat of *α*-2,6-Cl_2_-V under various magnetic fields. Temperature dependence of the total specific heat (*C*_p_) of *α*-2,6-Cl_2_-V below 1.0 K under various magnetic fields. At these low-temperatures, *C*_p_ can be regarded as that from magnetic contribution. For clarity, the *C*_p_ for 1.0, 2.0, 3.0, 4.0, and 5.0 T have been shifted upward by 1.0, 2.2, 3.3, 4.2, and 5.3 J/mol K, respectively. The black arrow at zero-field indicates the temperature of the phase transition to the ordered state. At 1.0 T, the phase transition disappears, and Schottky-like behavior appears at about 0.5 K. The red arrow shows a clear upturn in the 4.0 T data, suggesting some kind of field-induced phase transition at lower temperatures.

**Figure 4:**
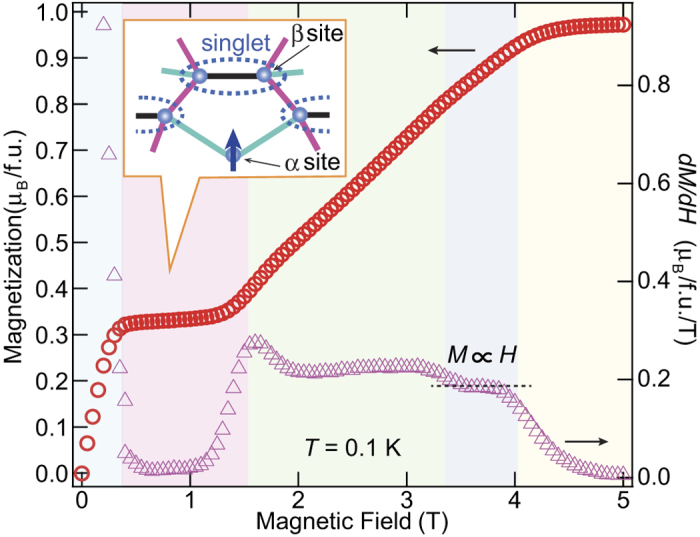
Low-temperature magnetization curve of *α*-2,6-Cl_2_-V. Magnetization curve and its field derivative for *α*-2,6-Cl_2_-V at 0.1 K. The illustration describes the predicted magnetic state in the 1/3 plateau phase, where spins on the *α* and *β* sites are fully polarized state and singlet dimer, respectively. The colored region between about 3.3 and 4.0 T indicates an unconventional phase with the relation of *H* ∝ *M* near the saturation field.

Figure 2(b) shows the temperature dependence of the total specific heat, *C*_p_, at zero-field. A clear broad peak appears at about 1.4 K, which is characteristic of a Schottky-like behavior associated with an energy gap between excited states. Consistent with such Schottky-like *C*_p_ behavior, a clear 1/3 magnetization plateau is observed from 0.4 to 1.3 T, indicating an energy gap between the excited states, as shown in Fig. 4. The six spins in the unit cell and the 1/3 magnetization plateau suggest the existence of a nonmagnetic singlet state with an excitation energy gap formed by the AFM interactions *J*_2_ and/or *J*_3_ between the *β* sites. If such is the case, residual *α*-site spins interact with one another through the excited states of the singlet state as discussed later. The magnetization curve indeed increases less steeply than the Brillouin function for free *S* = 1/2 spins up to the plateau phase, highlighting the presence of non-negligible AFM internal fields. In this context, a sharp peak of the specific heat observed at about 0.07 K is explained by a zero-field phase transition to long-range AFM order of the *α*-site spins. In the 1/3 plateau phase, these spins are fully polarized along the external field direction such that the ordered state disappears. The phase transition actually disappears in the specific heat at 1.0 T, while the Schottky-like behavior is shifted to around 0.5 K in response to the decrease of the energy gap, as shown in Fig. 3. In the case of the higher field region above the plateau phase, the field derivative of the magnetization curve (*dM*/*dH*) indicates linear behavior in *H* near the saturation field from 3.3 to 4.0 T, as shown in Fig. 4. This behavior is in clear contrast to the magnetization curve of conventional quantum spin systems, in which external field suppresses quantum fluctuations, which results in an upward curvature of the magnetization curve and a sharp *dH*/*dH* peak at the phase transition to fully polarized state^23^,^24^. The upturn observed in *C*_p_ at 4.0 T should therefore be attributed to unconventional magnetic behavior near the saturation field.

In order to identify the contribution of abovementioned AFM interaction, we assumed a simple situation in which the fully polarized *α*-site spin works only as an internal field 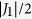 for each *β*-site spin in the high-field region above the 1/3 magnetization plateau. Accordingly, we considered a lattice system consisting of only *J*_2_ and *J*_3_ with an effective internal field 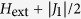, where *H*_ext_ is the external magnetic field. In the extreme case where 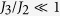, the two spins connected by the *J*_2_ interaction form a nonmagnetic singlet dimer with an excitation energy gap. In gapped cases such as this, the magnetization curve near the critical field—the end of the 1/3 plateau phase in the present case—increases with the square root of the applied field. For 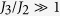 in contrast, the AFM interaction *J*_3_ forms a well-known *S* = 1/2 uniform AFM chain with a Tomonaga-Luttinger liquid ground state, and the energy gap disappears. The 1/3 plateau phase and sharp *dM*/*dH* peak that are observed here therefore indicate a singlet state arising from the AFM interaction *J*_2_ with 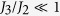, as described in Fig. 4. Accordingly, the effective interactions between the residual *α*-site spins are caused through the triplet excited states of the *J*_2_ singlet dimer^22^,^25^,^26^. They are roughly evaluated from the second-order and third-order perturbation treatment of the *J*_2_ term in the spin Hamiltonian (see Supplementary Information). We can consider that *α*-site spins form a uniform AFM chain with weak interchain interactions in the low-field region. These interactions should cause the phase transition to long-range AFM order of the *α*-site spins.

Finally, concerning the unconventional behavior near the saturation field, a plausible explanation is that this reflects a hidden order of spin multipoles caused by correlations between multi-magnon bound states, such as those in a spin-nematic phase^27^. The presence of such a phase is expected in the high-field region (near the saturation field) in *S* = 1/2 frustrated spin systems with FM interactions^27^,^28^, but has not been verified experimentally to date. In the PCPL, the three spins connected by the *J*_1_ FM interaction may stabilize the three-magnon bound state in the vicinity of the saturation field, resulting in multipole spin order. Additional experimental techniques, notably neutron scattering using deuterated samples, should afford a more quantitative description of this field-induced phase and clarify the pentagonal frustration effect.

## Methods

We synthesized 2,6-Cl_2_-V using a conventional procedure similar to that used for preparing the typical verdazyl radical 1,3,5-triphenylverdazyl^29^. The crystal structure was determined on the basis of intensity data collected using a Rigaku AFC-8R Mercury CCD RA-Micro7 diffractometer with Japan Thermal Engineering XR-HR10K. The magnetizations were measured using a commercial SQUID magnetometer (MPMS-XL, Quantum Design) and a capacitive Faraday magnetometer down to about 70 mK. The experimental results were corrected for diamagnetic contribution (−2.57 × 10^−4^), which is determined to become almost *χ*Τ = const. above about 200 K, and close to the value calculated by Pascal's method. The specific heat was measured using a hand-made apparatus by a standard adiabatic heat-pulse method down to about 50 mK. Considering the isotropic nature of organic radical systems, all experiments were performed using small randomly oriented single crystals. The *ab initio* MO calculations were performed using the UB3LYP method as broken-symmetry hybrid-density functional theory calculations. All the calculations were performed using the Gaussian 09 program package, and the basis functions used were 6–31G. To estimate the intermolecular magnetic interaction of the molecular pairs within 4.0 Å, we applied our previously presented evaluation scheme^30^.

## Additional Information

**How to cite this article**: Yamaguchi, H. *et al.* Experimental Realization of a Quantum Pentagonal Lattice. *Sci. Rep.* **5**, 15327; doi: 10.1038/srep15327 (2015).

## Supplementary Material

Supplementary Information
