# Supplementary material for: Experimental Realization of a Quantum Pentagonal Lattice
Source: Sci Rep. 2015 Oct 15;5:15327. doi: 10.1038/srep15327 (PMC4606929; doi:10.1038/srep15327)
Supplement: Supplementary Information [file srep15327-s1.doc]

**Experimental Realization of a Quantum Pentagonal Lattice**

H. Yamaguchi,* T. Okubo, S. Kittaka, T. Sakakibara, K. Araki, K. Iwase, N. Amaya,

T. Ono, Y. Hosokoshi*

*To whom correspondence should be addressed.

E-mail: yamaguchi@p.s.osakafu-u.ac.jp; yhoso@p.s.osakafu-u.ac.jp

1. **Crystal structure and intermolecular exchange interactions**

Figure S1 shows typical examples of the single crystals with the crystallographic axes indicated by diagonal lines. The crystallographic data obtained at 293 and 23 K are summarized in Table S1. The crystals contain two crystallographically independent molecules, M_1_ and M_2_, as shown in Fig. S1a. Significantly, neither is planar, and the R_1_-R_2_, R_1_-R_3_, and R_1_-R_4_ dihedral angles are about 28°, 49°, and 89° for M_1_, and 27°, 27°, and 81° for M_2_, respectively (R_1_, R_2_, R_3_, and R_4_ are defined in Fig. 1a). The M_2_ molecule has a carbon atom disorder because of its two-fold rotational symmetry in R_1_; thus, two carbon atoms are represented each with an occupancy of 0.5. Because this study focuses on the low-temperature magnetic properties, the crystallographic data at 23 K are used hereafter. The *ab initio* MO calculation indicates that more than 98% of the total spin density is present on R_1_∼R_3_ for both molecules. Therefore, the intermolecular interactions are mainly caused by short contacts of N or C related to the R_1_∼R_3_ rings. The evaluated dominant interactions *J*_1_, *J*_2_, and *J*_3_ correspond to interactions between molecular pairs in Figs. S2a, b, and c, respectively. The former interaction involves both crystallographically independent molecules, while the other two involve M_1_ only. There are three types of short intermolecular C-C contacts in *J*_1_, corresponding to distances of 3.43, 3.45, and 3.50 Å (Fig. S2a). A FM *J*_1_ was calculated for the two patterns of disordered carbon atoms in M_2_, and the average value was retained. Although the actual position of the disordered carbon atom in M_1_ may alternate, this should only affect *J*_1_ and should not substantially affect the magnetic ground state. The short C-C contact between the M_1_ molecules related to *J*_2_ is longer (3.64 Å, Fig. S2b) and is doubled by a two-fold rotation axis parallel to the *c* axis. The M_1_ molecular pair related to *J*_3_ has *d* glide reflection symmetry, where the glide plane is perpendicular to the *a* axis, and it has short N-C contacts of 3.51 Å (Fig. S2c). For this molecular pair, the small overlap of the *π* orbitals, which expand perpendicularly to the planes, owing to a relatively large dihedral angle between the related planes, should make the interaction weak.

These three interactions form a PCPL with a twisted *J*_1_-*J*_3_-*J*_2_-*J*_3_-*J*_1_ pentagonal unit (Fig. 1b)*.* To better understand the formation of this PCPL, this can be divided into two types of *J*_1_-*J*_1_-*J*_2_ chains along the *a*+*b* and *a*-*b* directions, as shown in Fig. S1d. These three-fold chains are connected by the interchain interaction *J*_3_, resulting in the formation of a PCPL with a unit cell containing six spins.


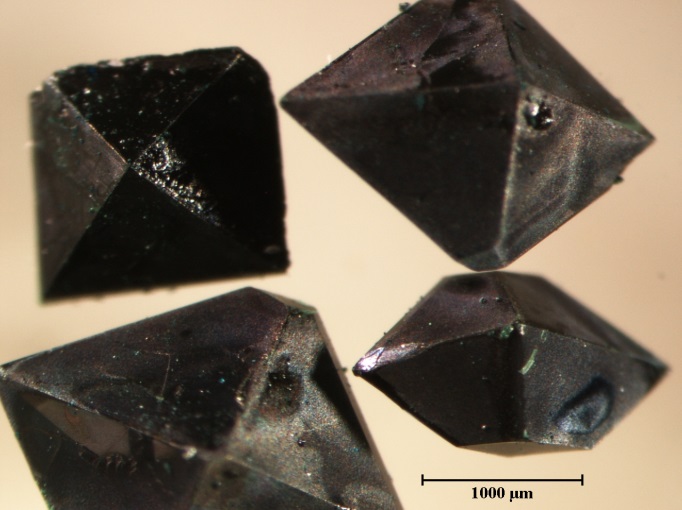


Figure S1: Single crystals of *α*-2,6-Cl_2_-V.

Table S1: Summary of crystallographic data for *α*-2,6-Cl_2_-V.

| Compound | *α*-2,6-Cl_2_-V | |
| --- | --- | --- |
| Formula | C_20_H_15_N_4_Cl_2_ | |
| Crystal system | Orthorhombic | |
| Space group | *Fdd*2 | |
| Temperature | 293(2) | 23(2) |
| *V*/Å^3^ | 11049.4(15) | 10586.1(12) |
| *a*/Å | 42.759(3) | 41.672(3) |
| *b*/Å | 15.5551(12) | 15.2563(10) |
| *c*/Å | 16.6127(13) | 16.6513(11) |
| *Z* | 24 | |
| *D*_calc_/g cm^-3^ | 1.379 | 1.439 |
| Total reflections | 4719 | 4631 |
| Reflection used | 4234 | 4558 |
| Parameters refined | 358 | |
| *R* [*I* > 2*σ*(*I*)] | 0.0346 | 0.0204 |
| *R_w_* [*I* > 2*σ*(*I*)] | 0.0846 | 0.0499 |
| Goodness of fit | 1.081 | 1.054 |
| CCDC | 1403881 | 1403882 |


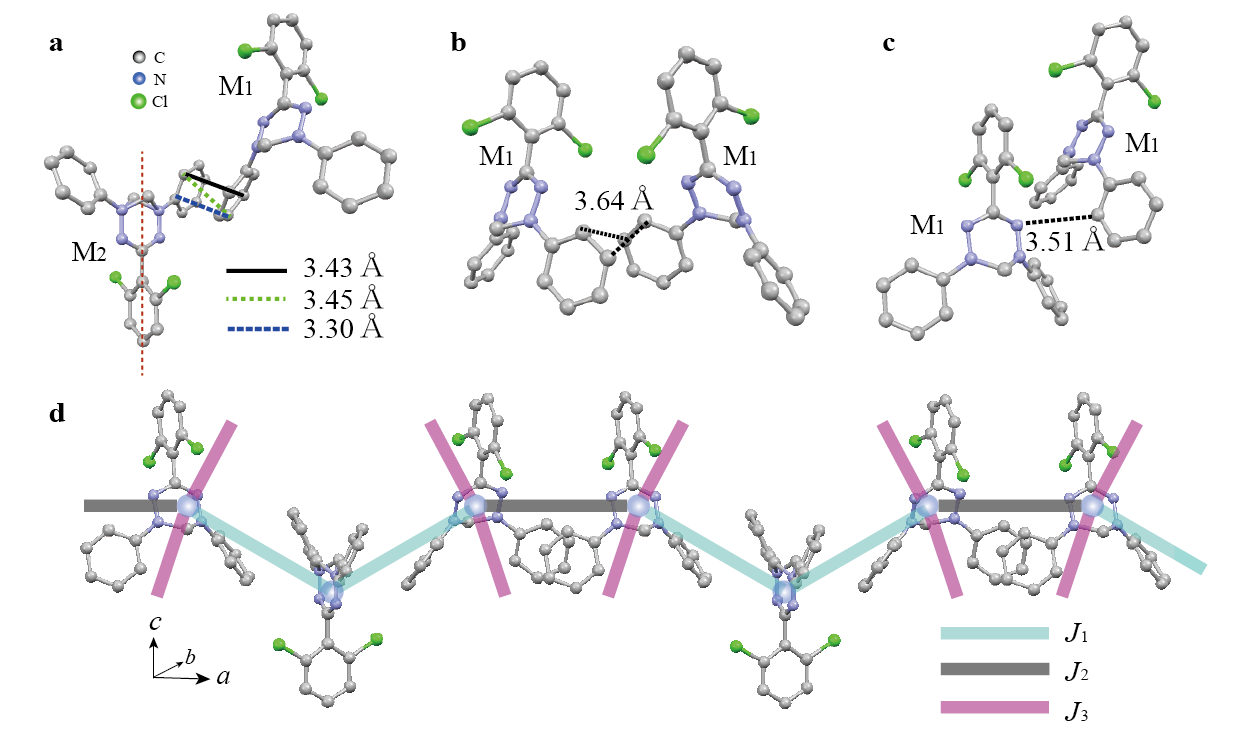


Figure S2: Crystal structure of *α*-2,6-Cl_2_-V. M_1_ and M_2_ are crystallographically independent molecules. The dotted red line in the center of M_2_ denotes the two-fold rotation axis. Molecular pairs associated with the intermolecular magnetic interactions (**a**) *J*_1_, (**b**) *J*_2_, and (**c**) *J*_3_, respectively. **d**, Molecular arrangement forming a *J*_1_- *J*_1_- *J*_2_ threefold chain along the *a*+*b* direction.

1. **Higher-order interactions between *α*-site spins**

In the low-field region below the 1/3 magnetization plateau phase, a singlet dimer with an excitation energy gap is formed by the AFM interaction *J*_2_ between the *β*-site spins. If such is the case, the residual *α*-site spins interact with one another through the triplet excited states of the *J*_2_ singlet dimer. The effective interactions are evaluated from the higher-order perturbation treatment of the *J*_2_ term in the spin Hamiltonian. If we consider up to third-order perturbation treatment of the *J*_2_ term, there are four types of exchange paths, which correspond to interactions between 1-2, 1-3, 1-4, and 1-5 spins, as shown in Fig. S3. The interaction between 1-3 spins has two different patterns, *J*_1_-*J*_2_-*J*_3_-*J*_1_ and *J*_1_-*J*_3_-*J*_1_, which induce FM and AFM interactions with the same absolute value, respectively, and cancel each other. The interaction related to 1-2 spins is derived from the second-order perturbation treatment and form a uniform AFM chain. The interaction related to 1-4 and 1-5 are derived from the third-order perturbation treatment and correspond to a weak AFM and FM interchain interaction with the same absolute value. Assuming the evaluated interactions from the MO calculations, the intra- and interchain interactions are given by *J*_1_^2^/2*J*_2_~0.33 K and |*J*_1_^2^*J*_3_/4*J*_2_^2^|~0.13 K, respectively. Although the actual values should be less than those evaluations owing to the relation *J*_3_/*J*_2_<<1, these interactions should cause the phase transition to the long-range AFM order of the α-site spins.


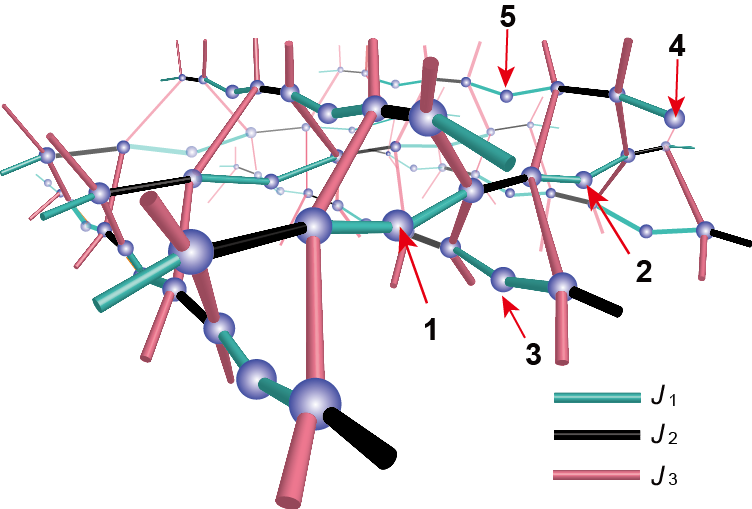


Figure S3: Partially corner-shared pentagonal lattice (PCPL) of the α-2,6-Cl_2_-V crystal. The blue sphere and the three types of solid lines denote spin-1/2 and intermolecular interactions *J*_1_, *J*_2_, and *J*_3_, respectively.
